# A scoping review of genetic studies of treatment-resistant schizophrenia

**DOI:** 10.3389/fgene.2026.1802472

**Published:** 2026-05-20

**Authors:** Urmi Das, Hayley Riel, Monserrat Rocio Enriquez, Mina Shirinbakhshmasoleh, Allison Rose, Marc Herrera, Yi Lu, Kaarina Kowalec

**Affiliations:** 1 College of Pharmacy, University of Manitoba, Winnipeg, MB, Canada; 2 Department of Medical Epidemiology and Biostatistics, Karolinska Institutet, Stockholm, Sweden; 3 Department of Biochemistry and Medical Genetics, University of Manitoba, Winnipeg, MB, Canada

**Keywords:** clozapine, genetics, pharmacogenomics, polygenic risk score, schizophrenia, treatment-resistant schizophrenia

## Abstract

**Background:**

This scoping review synthesized the current evidence on the genetic architecture of treatment-resistant schizophrenia (TRS). TRS is typically characterized by an inadequate response to antipsychotic treatment in individuals with schizophrenia. Approximately 30% of individuals with schizophrenia develop treatment resistance, which is associated with greater disability, poorer prognosis, and increased mortality compared to treatment-responsive schizophrenia. Numerous studies have explored various genetic aspects of TRS; therefore, a scoping review was needed to summarize findings and consistent patterns, clarify the current level of understanding, and highlight remaining knowledge gaps.

**Methods:**

A systematic search was conducted in PubMed up to March 2025. This scoping review followed PRISMA-ScR guidelines. Studies were included if they reported on genetic factors of TRS and its related constructs (clozapine resistance). Data on treatment resistance, study design, population characteristics, and genetic findings were extracted and synthesized.

**Results:**

A total of 102 studies were included. Definitions of TRS varied across studies, with most using proxies such as clozapine use, antipsychotic polypharmacy, or they used presence of symptoms despite antipsychotic treatment. Most studies compared TRS with treatment-responsive schizophrenia and predominately included participants of European genetic ancestry. Genetic findings spanned common variants (e.g., identified from genome-wide association studies or in cumulative measures such as polygenic risk scores [PRS]), rare variants, and copy number variants and functional genomics such as gene expression and epigenetic markers. Common variants dominated the literature but explained only a small proportion of TRS liability. Higher schizophrenia PRS, specific rare variants, and copy number variants were associated with TRS, while TRS-specific PRS remain in development. GWAS largely focused on schizophrenia broadly, with substantial genetic overlap between TRS and schizophrenia. Transcriptomic and epigenomic data provide additional but limited insights, often confounded by drug exposure.

**Conclusion:**

Heterogeneous TRS definitions and limited ancestry diversity constrain progress, and robust TRS-specific genetic markers remain scarce. Harmonized criteria and larger, diverse cohorts are needed. Integrating genetic, epigenetic, and clinical data could improve early risk identification and guide precision treatment strategies.

## 1 Introduction

Schizophrenia is a psychiatric disorder affecting ∼1% of the global population (Solmi et al., 2023; Saha et al., 2005). This illness is characterized by positive symptoms (e.g., hallucinations, delusions), negative symptoms (e.g., social withdrawal, blunted affect), and deficits in cognition (e.g., impairments in attention, memory, and executive function). These symptoms can substantially impair daily functioning and often require lifelong management with antipsychotic medications, psychotherapy, and support services. Approximately 30% of individuals with schizophrenia do not respond to ≥2 antipsychotic medication despite multiple trials at adequate dose and duration (Kane et al., 1988; Conley and Kelly, 2001). This subset of individuals is classified as having treatment-resistant schizophrenia (TRS). TRS is associated with significant clinical and societal burden, including more frequent hospitalizations (Kennedy et al., 2014), increased risk of suicide and mortality (Wimberley et al., 2017; Wimberley et al., 2016), greater functional impairment (Millgate et al., 2022), and reduced quality of life (Kennedy et al., 2014) compared to treatment-responsive schizophrenia. Clozapine is currently the only antipsychotic specifically approved for TRS, and while it is effective at reducing hospitalizations and suicide rates (Land et al., 2017; Masuda et al., 2019; Stroup et al., 2016), it is also associated with strong adverse effects and requires rigorous blood monitoring (Tiihonen et al., 2017).

Schizophrenia is highly heritable, with pedigree-based estimates from family and twin studies of approximately 70%–80% (Sullivan et al., 2003). Of this genetic liability, approximately 25% is explained by common genetic variation in European populations, with 287 common risk loci identified to date, implicating synaptic, neuronal, and immune pathways (Liu et al., 2017; The Network and Pathway Analysis Subgroup of the Psychiatric Genomics Consortium, 2015; Trubetskoy et al., 2022). The genetics of treatment response in schizophrenia are likely related, but not identical, to the genetics of schizophrenia risk itself, as this has been observed in other psychiatric disorders such as depression (Xiong et al., 2025). Genetics research on TRS has employed a range of approaches, including common and rare variants, and copy number variants (CNV), and functional genomic investigations. Given the heterogeneity of schizophrenia and the diversity of methods and phenotypes used in TRS research, a synthesis of the genetic findings is needed to clarify what is known about the genetic architecture of TRS and to identify key knowledge gaps. A better understanding of the genetic architecture that contributes to poor treatment response in schizophrenia remains an important unmet need and could facilitate earlier identification of individuals at risk for TRS, support earlier or optimized interventions (e.g., clozapine initiation), and aid in the identification of novel pharmacological targets that may not be apparent when examining schizophrenia as a single, homogeneous entity.

We conducted a scoping review to summarize and assess the current literature on the genetic architecture of TRS and related outcomes, including treatment response and drug reactions, particularly to clozapine. More specifically, we aimed to 1) Examine how TRS has been operationally defined across genetic studies; 2) Synthesize the genetic findings for TRS; 3) Identify potential genetic factors associated with TRS; and 4) Summarize the current state of knowledge on TRS genetics and highlight unexplored areas. Conducting a scoping review enabled a broad exploration of the genetic underpinnings of TRS by incorporating studies focusing directly on TRS as well as those assessing treatment outcomes with clozapine. By consolidating these findings, our goal is to provide an overview of the current landscape of TRS genetics and contribute to an improved mechanistic understanding of treatment resistance within schizophrenia.

## 2 Materials and methods

### 2.1 Search strategy

The study protocol was registered on the Open Science Framework (registration doi: 10.17605/OSF.IO/938BR). Articles were identified through PubMed up to and including March 2025. PubMed was specifically chosen due to its comprehensive coverage of genetic and psychiatric research. The following search query was used: (“genetic” [All Fields] OR “genetical” [All Fields] OR “genetically” [All Fields] OR “genetics” [MeSH Subheading] OR “genetics” [All Fields] OR “genetics” [MeSH Terms]) AND (“schizophrenia, treatment resistant” [MeSH Terms] OR (“schizophrenia” [All Fields] AND “treatment resistant” [All Fields]) OR “treatment-resistant schizophrenia” [All Fields] OR (“treatment” [All Fields] AND “resistant” [All Fields] AND “schizophrenia” [All Fields]) OR “treatment resistant schizophrenia” [All Fields]). In addition to the database search, the reference lists of included articles were screened for additional relevant publications that were not captured in the initial search. The scoping review was conducted in accordance with the Preferred Reporting Items for Systematic Reviews: Extension for Scoping Reviews (PRISMA-ScR) (Tricco et al., 2018).

### 2.2 Study selection and data extraction

Article screening was performed using Covidence and involved initial title and abstract screening, full-text review, and data extraction. Screening was conducted by two independent reviewers, and any discrepancies were resolved by a third independent reviewer.

Articles were included if they reported on the underlying genetic components or architecture of TRS, included participants aged 18+, and were published in English language. Review articles, grey literature (e.g., preprints), theses or dissertations, case reports, and non-article publications were excluded. For each included study, data were extracted on study design, TRS definition, population characteristics, and genetic findings.

## 3 Results

**FIGURE 1:**
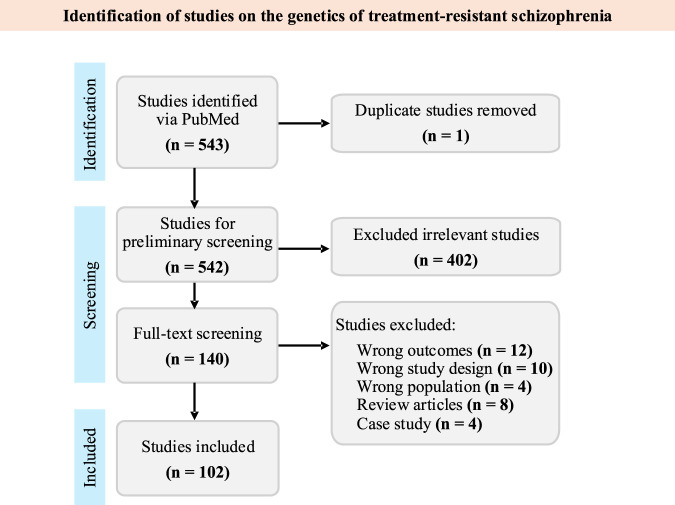
PRISMA Diagram for the scoping review of the genetic architecture of treatment-resistant schizophrenia.

**FIGURE 2:**
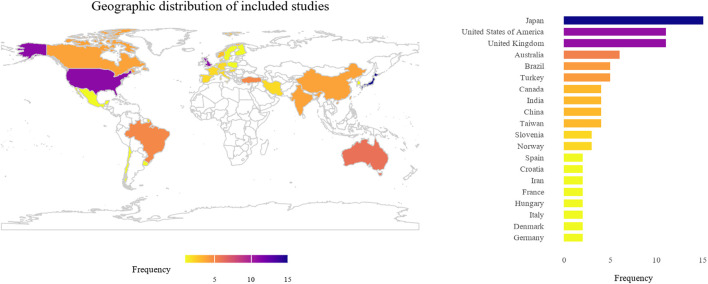
Global distribution of included studies. The world map (left) illustrates the geographic distribution of studies included in the review, with color intensity indicating the number of studies per country. The accompanying bar plot (right) displays the corresponding frequencies by country.

**FIGURE 3:**
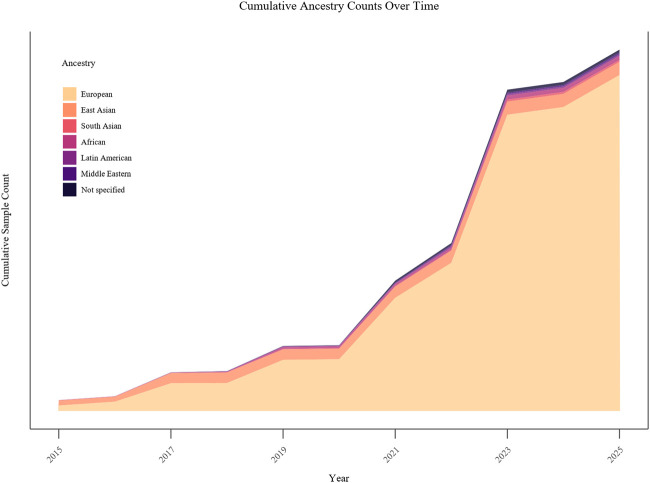
Cumulative ancestry-specific sample counts over time. The plot shows cumulative sample counts from included studies by ancestry between 2015 and 2025, with the x-axis representing publication year and the y-axis representing cumulative sample size.

A total of 543 articles were identified through PubMed (Figure 1). After removing duplicates and applying the screening protocol, 102 articles were included in the scoping review (Supplementary Table S1). Most of the studies were from Japan (*n* = 15), followed by United Kingdom (*n* = 11), and United States of America (*n* = 11, Figure 2). In terms of population structure (Figure 3), study participants were predominantly of European descent (*n* = 47 studies, 46%), followed by East Asian (24%), Middle Eastern (8%), Latin American (5%), South Asian (4%), and African (1%). Additionally, 12% of studies included multi-ancestry cohorts; however, even within these populations, European ancestry was the majority comprising 45%–90% of participants.

The comparator groups varied slightly: the majority (*n* = 48, 69%) included individuals without TRS (non-TRS or treatment-responsive cases) as the comparator, and the remaining 21 studies (31%) specified healthy controls as a comparator, of which 10 also included non-TRS cases as a second comparator.

### 3.1 Defining treatment-resistant schizophrenia (TRS)

There was considerable variability in how TRS was defined across studies. Seven broad definition categories were identified: 1) Failure to respond to two or more antipsychotics (*n* = 27); 2) Continued worsening of symptoms despite antipsychotic treatment (*n* = 21); 3) Clozapine use (*n* = 30); 4) Polypharmacy use (*n* = 6); 5) Worsening symptoms and clozapine use (*n* = 5); 6) Worsening symptoms and polypharmacy use (*n* = 4); or 7) Worsening symptoms and a failure to respond to antipsychotics (*n* = 9). Approximately 30% (*n* = 29/102) incorporated clinical rating scales (e.g., Positive and Negative Syndrome Scale, Global Assessment of Functioning) to support their identification of TRS.

A subset of studies (*n* = 5) examined clozapine-resistance, often referred to as “ultra-resistant” (Mouaffak et al., 2011a) or “super-refractory” (Krzystanek et al., 2021) schizophrenia. Unlike TRS as defined above, ultra-resistant TRS was characterized by non-response to clozapine as defined by adequate duration (≥6 weeks to 6 months) and therapeutic dosing of clozapine (250–600 mg/day). Several studies also incorporated clozapine plasma concentration thresholds (≥350 ng/mL) or clozapine-to-norclozapine metabolic ratios as part of the definition, in addition to persistent positive symptoms assessed with clinical rating scales.

### 3.2 Genetic insights

The genetic findings related to TRS were grouped into three domains (Table 1): common variant studies (*n* = 75) (Li and Meltzer, 2014; Miyazawa et al., 2022; Naveen et al., 2020; Ji et al., 2008a; Hong et al., 2003; Teo et al., 2012; Tsai et al., 2001; Mouaffak et al., 2011b; Taheri et al., 2023; Jia et al., 2011; Takao et al., 2006; Escamilla et al., 2018; Hotta et al., 2011; Hwang et al., 2005; Hong et al., 2000; Bozina et al., 2008; Hajj et al., 2019; Willcocks et al., 2021; Kondo et al., 2003; Bosia et al., 2015; Piatkov et al., 2017; Lim et al., 2023; Ota et al., 2012; Arranz et al., 1998; Rajagopal et al., 2018; Taylor et al., 2016; Terzić et al., 2016; Terzić et al., 2015a; Zhang et al., 2013; Legge et al., 2017; Pinheiro et al., 2017; Xu et al., 2015; Konte et al., 2021; Frank et al., 2015; Werner et al., 2020; Terzić et al., 2015b; Souza et al., 2010; Kogure et al., 2021; Pae et al., 2006; Del Casale et al., 2026; Aytac et al., 2021; Kohlrausch et al., 2008; Eap et al., 2004; NCBI, 2026a; Rajkumar et al., 2012; Nakata et al., 2021; Taylor et al., 2017; Ruderfer et al., 2016; O’Connell et al., 2023; Zazueta et al., 2022; NCBI, 2026b; Patil et al., 2021; Inada et al., 2003; Ji et al., 2008b; Gasse et al., 2019; Facal and Costas, 2025; Talarico et al., 2022; Bani-Fatemi et al., 2019; Ozdemir et al., 2025; Cheng et al., 2023; Griffiths et al., 2023; Bilic et al., 2014; Aytac et al., 2022; Oishi et al., 2018) including genome-wide association studies (GWAS), candidate genes, polygenic risk scores (PRS); rare variant and CNVs (*n* = 10) (Legge et al., 2017; Ruderfer et al., 2016; Farrell et al., 2023; Morimoto et al., 2021; Räsänen et al., 2025); and functional genomics studies (*n* = 17) (Ozdemir et al., 2025; Räsänen et al., 2025; Funahashi et al., 2023; Menus et al., 2020; Moretti et al., 2018; Nakaz et al., 2017; Pérez-Rodríguez et al., 2023; You et al., 2020), which characterized how genetic variation influences cellular and molecular processes, including gene expression and epigenetic mechanisms. Common genetic variant studies were defined as those assessing variants with a minor allele frequency (MAF) of ≥5% of the population, whereas rare variants were defined as those with MAF <1%; however, these categories were not distinct.

**TABLE 1:** Summary of genetic findings related to treatment-resistant schizophrenia (TRS).

Category	Summary
Common variant - candidate genes	Studies examined common genetic variants related to neurotransmitter signaling and antipsychotic drug metabolism in relation to TRS. Variants in dopaminergic, serotonergic, and glutamatergic pathway genes were associated with altered receptor function, neurotransmission, and antipsychotic response, including clozapine efficacy, while some suggested sex-specific effects. Variation in clozapine metabolism genes, particularly CYP enzymes, as well as safety-associated variants such as *ACKR1*, may contribute to variability in drug levels, treatment response, adverse effects, and the need for individualized prescribing
Common variant - genome-wide association studies	GWAS examined broad TRS-related phenotypes, including TRS case-control status, symptom severity in clozapine-treated individuals, clozapine plasma concentrations, and clozapine-induced neutropenia, with most conducted in ancestry-specific cohorts and few using multi-ancestry samples. The largest GWAS found TRS and non-TRS schizophrenia were highly genetically correlated but identified no TRS-specific loci. GWAS of clozapine pharmacokinetics and neutropenia more consistently identified significant loci, particularly in CYP and UGT metabolism genes and in ancestry-relevant neutropenia-associated loci, highlighting the importance of pharmacogenomics and ancestry in clozapine-related outcomes
Common variant - polygenic (risk) scores (PRS)	Studies show a higher schizophrenia PRS is modestly associated with TRS and influences clozapine use and response, with elevations in schizophrenia PRS may be linked to better clozapine outcomes, while the highest PRS levels tend to occur in individuals with the most severe and refractory illness. PRS for other traits show little association with TRS, though glutamatergic PRS may modify the impact of childhood adversity on cognitive outcomes in TRS.
Rare variants and copy number variants	Studies showed that TRS is associated with a higher burden of rare genetic variants than non-TRS. TRS individuals carry more rare damaging variants and CNVs, including schizophrenia- and neurodevelopment-associated CNVs. Variants affecting drug metabolism and transport genes may also influence antipsychotic response, suggesting a genetic contribution to treatment resistance
Functional genomics - gene expression	TRS showed distinct gene expression patterns compared with non-TRS and healthy controls, indicating biological differences as well as variability in study design and treatment exposure. Dysregulation primarily involved genes related to stress response, synaptic function, neurodevelopment, immune signaling, and regulatory non-coding RNAs. Several transcriptomic changes appeared influenced by antipsychotic treatment, particularly clozapine, affecting miRNAs, lncRNAs, and immune markers. Overall, these findings suggested that TRS comprised altered molecular pathways linked to synaptic regulation, neurodevelopment, and treatment response, although some effects may reflect underlying schizophrenia biology rather than treatment resistance alone
Functional genomics - epigenetic	Studies primarily reported DNA methylation changes in TRS. While some studies found no significant differences, others reported methylation patterns that distinguish TRS or predict clozapine exposure. Twin studies further suggested that clozapine itself can modify methylation. Overall, it remains unclear whether these changes reflect TRS biology or medication effects

#### 3.2.1 Common genetic variant studies

##### 3.2.1.1 Candidate genes

Fifty-eight studies examined associations between specific common genetic variants and TRS, with a focus on neurotransmitter systems (dopaminergic or serotonergic) or antipsychotic drug metabolism.

Variants in dopaminergic genes, including *DRD1*, *DRD2*, *DRD3*, *DRD4*, *DAT*, and *DDC*, were frequently linked to altered receptor function and dopaminergic signaling, contributing to diminished efficacy of dopamine-targeting treatments, including clozapine. The *DRD3* rs6280 (Ser9Gly) polymorphism was associated with clozapine resistance in TRS, while several *DRD2* SNPs were associated with variability in antipsychotic response (Casale et al., 2023). Variants in *COMT*, a key the dopamine-metabolizing gene that influences enzymatic activity, were linked to TRS. In females with TRS, low-activity *COMT* genotypes were more frequent, whereas high-activity *COMT* haplotypes were more common in treatment responders, suggesting a protective effect. In contrast, males with TRS showed a higher frequency of the Met allele at rs4680, particularly the Met/Met genotype, indicating that although reduced *COMT* activity is associated with TRS in both sexes, the specific genotype patterns differ between males and females (Sagud et al., 2018).

Genes in the serotonergic pathway, including *HTR3A*, *HTR3B*, *HTR2A*, *HTR4*, *5-HT1A*, *SLC6A4* (serotonin transporter), *SERT-PR*, *SERT-in2*, and *5-HT6* were frequently associated with TRS. Variants in these genes were found to disrupt the serotonin-dopamine balance critical for optimal antipsychotic efficacy and clozapine response. Similarly, glutamatergic genes such as *GRIN1*, *GRIN2A*, *GRIN2B*, *SLC1A2*, *SLC6A9*, *GRIA1*, *GRM2*, and *GAD1*, were identified in TRS in relation to cognitive deficits and dysregulation of excitatory and inhibitory neurotransmission.

Common variants in cytochrome P450 (CYP) enzymes such as *CYP1A2*, *CYP3A4*, *CYP3A5*, and *POR* (cytochrome P450 oxidoreductase), which play central roles in clozapine biotransformation (Demirbugen et al., 2023), were shown to influence plasma drug concentrations and therapeutic efficacy, contributing to inter- and intra-individual variability in treatment response in TRS (Demirbugen et al., 2023). Individuals who carried multiple rare damaging variants or clinically significant CNVs in *CYP1A2* exhibited subtherapeutic clozapine responses, altered clozapine pharmacokinetics, and required higher clozapine doses (Kappel et al., 2024). Additionally, variants in *CYP2C18*, *CYP2C19*, and of *UGT2* gene family were shown to influence the clozapine-to-norclozapine metabolic ratio, a clinically relevant marker for enzymatic activity and individualized dose adjustment (Okhuijsen-Pfeifer et al., 2022).

Beyond metabolism and efficacy, rs2814778 emerged as a safety-related pharmacogenetic variant. Individuals of African ancestry carrying this variant were associated with clozapine-induced neutropenia (odds ratio [OR] = 20.4, *p* = 3.44 × 10^−7^) and explained more variance in neutrophil counts than genetic ancestry alone, making it a key marker for ancestry-informed safe prescribing (Legge et al., 2019). In addition, genetic predispositions interacted with lifestyle factors (e.g., smoking and caffeine intake), influencing variability in clozapine metabolism and response.

##### 3.2.1.2 Genome-wide association studies (GWAS)

Eight GWAS investigated broad aspects of TRS such as case-control status, symptom severity among clozapine-treated individuals, clozapine plasma concentrations, or clozapine-induced neutropenia. Two GWAS used multi-ancestry cohorts, and the remaining were conducted in ancestry-specific samples (four European, one African, and one Turkish cohort).

The largest included GWAS analyzed 10,501 TRS cases and 24,542 healthy controls, alongside a second GWAS of 20,325 non-TRS cases and 30,122 healthy controls (Pardiñas et al., 2022). Interaction analyses comparing effect sizes between TRS and non-TRS revealed a high genetic correlation (r = 0.966) and replicated known schizophrenia loci, but no TRS-specific associations were detected; SNP-based heritability for TRS was estimated at 1%–4% (Pardiñas et al., 2022). A smaller separate GWAS (478 TRS vs. 808 non-TRS cases) reported a suggestive association at rs79229764 within *LINC00523* (*p* = 1.8 × 10^−7^) (Lenk et al., 2024).

Five GWAS concentrated on clozapine treatment in TRS. Focusing on symptom severity among clozapine-treated schizophrenia individuals, genome-wide analyses of 684 TRS cases from five cohorts reported a suggestive association between symptom severity and a variant in *NFIB* (*p* = 3.78 × 10^−7^) (Okhuijsen-Pfeifer et al., 2022). In relation to clozapine metabolism, a GWAS using 10,353 pharmacokinetic assays from 2,989 TRS cases (with >90% probability of European ancestry) identified four genome-wide significant loci in *CYP* and *UGT*, drug metabolism genes associated with clozapine and norclozapine plasma levels (Pardiñas et al., 2019). A subsequent cross-ancestry GWAS using 16,068 pharmacokinetic assays from 4,495 TRS cases revealed ancestry-specific differences in clozapine metabolism and identified eight associated loci, most of which had stronger effects in non-European populations including Southwest Asian, North African, Sub-Saharan African (Pardiñas et al., 2023).

Genome-wide studies on clozapine-induced neutropenia identified key genetic associations, while not reflecting TRS biology *per se*, provide clinically valuable insight into susceptibility of this adverse effect. One study of 66 neutropenia cases (absolute neutrophil count [ANC] ≤1,500/mm^3^) and 5,583 clozapine treated controls (maintained ANC ≥ 2,000/mm^3^ for at least 1 year of clozapine treatment) of European ancestry identified genetic variation in hepatic transporter genes *SLCO1B3* and *SLCO1B7* (OR = 4.32, *p* = 1.79 × 10^−8^), while also replicating the previously reported *HLA-DQB1* variant (OR = 15.6, *p* = 0.015, positive predictive value = 35.1%) (Konte et al., 2021). Another GWAS of lowest ANC during clozapine treatment in 552 individuals of African ancestry identified two loci associated with low neutrophil count, with the strongest association observed at rs2814778 in *ACKR1* (*p* = 4.21 × 10^−21^) (Legge et al., 2019).

Lastly, one GWAS of 84 individuals (31 TRS, 53 non-TRS) tested 1,178,234 SNPs using a main effect model for TRS status and an interaction model with childhood trauma. However, no associations reached genome-wide significance in either model (Koga et al., 2017).

##### 3.2.1.3 Polygenic risk scores (PRS)

Eleven studies examined schizophrenia PRS in relation to TRS given the hypothesis that higher genetic burden for schizophrenia may increase the risk of treatment resistance. In a large study of 10,501 TRS cases and 20,325 non-TRS cases, schizophrenia PRS explained ∼2% of the variance in TRS liability, indicating a modest but significant association (Pardiñas et al., 2022). Several studies also assessed the relationship between schizophrenia PRS and clozapine treatment outcomes. Higher schizophrenia PRS was associated with greater clozapine dose requirements in large UK cohorts such as *CLOZUK2* and *CLOZUK3* (approximately 4,000 individuals), as well as in a Norwegian cohort of 417 individuals (Kappel et al., 2023). Among TRS individuals receiving clozapine, those in the highest schizophrenia PRS tertile were 1.94 times more likely to exhibit low symptom severity than those in the lowest tertile *(p* = 6.84 × 10^−4^), indicating a better response to clozapine in this subgroup (Okhuijsen-Pfeifer et al., 2022). In contrast, another study found that although individuals treated with clozapine had higher schizophrenia PRS *(p =* 0.02) than those who had never received clozapine, the highest schizophrenia PRS were observed among clozapine non-responders or extreme-TRS cases, particularly those with poor premorbid functioning, early illness onset, and severe illness progression (Okhuijsen-Pfeifer et al., 2022). The findings reflect that moderately elevated schizophrenia PRS may be associated with better clozapine response within TRS cohorts, whereas the highest PRS values tend to cluster in individuals with the most severe, and treatment-refractory illness.

Three studies have investigated PRS for non-schizophrenia traits in relation to TRS. A large population-based Swedish study (*n* = 2,997 TRS; *n* = 3,675 non-TRS) conducted by members of our group examined PRS for bipolar disorder, major depressive disorder, autism spectrum disorder, cognitive ability, and educational attainment, but found no significant associations with TRS (Kowalec et al., 2021). Similarly, another study assessed PRS for BMI, smoking behaviour, caffeine intake, and a range of psychiatric, cognitive, and personality traits in relation to clozapine dose among individuals with TRS (*n* = 4,459), and likewise reported no significant association (Kappel et al., 2023). The third study derived a glutamatergic PRS from variants in glutamate signaling genes in association with childhood adversity and IQ in TRS (*n* = 51) and non-TRS (*n* = 154). Although individuals with TRS had a slightly higher mean glutamatergic PRS compared to non-TRS, this difference was not statistically significant (Mohamed Saini et al., 2023). However, glutamatergic PRS significantly moderated the impact of childhood adversity on cognitive and structural outcomes in TRS, amplifying the negative association with IQ (β = −125.05*, p* = 0.0002), whereas no such moderation was observed in non-TRS.

#### 3.2.2 Rare genetic variants and copy number variants (CNV)

A study of 112 individuals identified as having severe TRS (defined by a minimum of 5 years of continuous hospitalization) reported significantly greater enrichment of rare damaging variants in intolerant genes (loss-of-function variants OR = 1.91, missense variants OR = 2.90) compared to 218 non-TRS cases and 4,929 healthy controls (Zoghbi et al., 2021). Nearly half (48.2%) of the individuals with severe TRS carried at least one rare damaging variant compared to 29.8% and 25.4% in non-TRS individuals and healthy controls, respectively.

Multiple studies reported a higher burden of CNVs among individuals with TRS. One such study found a significant association between total genome-wide copy number duplication burden and treatment resistance (OR = 1.04*; p* < 0.01) between TRS and non-TRS cases (Martin and Mowry, 2016). A large study comparing TRS (n = 509) and non-TRS (*n* = 21,094) cases found that rare CNVs were significantly more prevalent in TRS–approximately 4% in TRS cases compared to 2.18% in non-TRS cases. In TRS cases, 9.2% had at least one CNV linked to neuropsychiatric risk and 4.7% carried CNVs associated with neurodevelopmental risk, although these CNVs are also enriched in schizophrenia in general, making their TRS-specificity uncertain. The most frequently reported CNVs included duplications at 15q11.2–q13.1 and 16p11.2, and deletions at 22q11.21 (Kushima et al., 2017). In addition, somatic CNVs were found at higher frequencies in TRS compared to non-TRS individuals *(p* = 0.03), affecting transporter gene *ABCB11* associated with antipsychotic metabolism (Maury et al., 2023). One final study of TRS cases reported finding rare damaging variants, or clinically significant CNVs, in *CYP1A2,* which altered clozapine pharmacokinetics (Kappel et al., 2024).

#### 3.2.3 Functional genomic research

##### 3.2.3.1 Gene expression

Across many studies, TRS was associated with distinct gene expression profiles in peripheral blood and post-mortem brain tissue compared to non-TRS and healthy controls. The direction and magnitude of effects varied likely reflecting heterogeneity in TRS definitions and study designs. These transcriptional alterations were modulated by regulatory elements, including microRNA (miRNAs) and long non-coding RNAs (lncRNAs), particularly in the context of antipsychotic exposure with clozapine.

Transcriptomic studies highlighted dysregulation of stress-related genes (e.g., *SIRT1*, *TRIM28*) and synaptic regulators (e.g., *CNR1*, *UFD1L*, *AKT1*, *DICER1*) in TRS compared to non-TRS cases (Pérez-Rodríguez et al., 2023). In monozygotic twins discordant for clozapine response, protocadherin gene alterations in iPSC-derived neurons were identified, implicating synaptic remodeling in clozapine efficacy (Nakaz et al., 2017). Several schizophrenia-related miRNAs were upregulated in TRS relative to non-TRS, with miR-181b-5p, miR-195-5p, and miR-301a-3p consistently dysregulated and associated with treatment response via silencing of antipsychotic receptor genes (Alacam et al., 2016). Additional candidates include clozapine-regulated miR-675-3p, linked to neuronal function via plasma exosomes, as well as miR-218-5p and miR-1262, which have been reported as early blood-based biomarkers for TRS, and miR-143-3p, induced by NRG1/mTOR signaling, as a putative mediator of olanzapine response (Sun et al., 2022).

*BDNF*, a critical neurodevelopmental regulatory gene, showed region-specific expression changes in brain and variable serum levels based on antipsychotic treatment type (Badrlou et al., 2021). Similarly, elevated expression of *BDNF* and associated lncRNAs (e.g., *BDNF-AS*, *MIR137HG*, *MIAT*, *PNKY*) were reported in individuals with TRS compared to healthy controls, which more so highlight schizophrenia biology and not necessarily that of treatment resistance (Badrlou et al., 2021). Another important neurodevelopmental and synaptic function, the NRG–ErbB signaling pathway, showed increased transcript expression in TRS; particularly, higher *P70S6K* mRNA levels (*p*_corrected_ = 0.018) were negatively correlated with illness duration (Mostaid et al., 2017). The last study found peripheral *TNF-α* mRNA level reductions in TRS (*p <* 0.001) compared to healthy controls. The levels increased with clozapine treatment; however, as the comparison was made with healthy controls, the contribution of underlying schizophrenia biology to these findings cannot be completely excluded (Mostaid et al., 2018; Kluge et al., 2009).

##### 3.2.3.2 Epigenetics

DNA methylation was a focus for multiple studies. One genome-wide analysis found no significant methylation sites between TRS (*n* = 43) and non-TRS (*n* = 66) individuals (De Luca et al., 2023). In contrast, another study using a two-phase (discovery and validation) design identified six probes that distinguished TRS (*n* = 48) from non-TRS (*n* = 48) individuals, with a methylation risk model achieving 88.3% accuracy (area under the curve = 0.95) (Lu et al., 2023).

An epigenome-wide study (EWAS) of >431,000 methylation sites identified seven differentially methylated positions associated with clozapine exposure in TRS *(p <* 9 × 10^−8^). These sites showed a mean methylation difference of 1.47% (SD = 0.24%), with hypermethylation observed only in TRS individuals *(p =* 0.0156) (Hannon et al., 2021) compared to non-TRS individuals. Another EWAS comparing TRS (*n* = 67) with non-TRS individuals not treated with clozapine (*n* = 314) generated an epigenetic score associated with clozapine use (R^2^ = 0.022, *p* = 3.85 × 10^−3^) (Ki et al., 2024). Lastly, a study of monozygotic twins with TRS but discordant for clozapine response reported methylation differences between responders and non-responders. Clozapine responders showed a higher proportion of differentially methylated genes related to neuronal and synaptic functions (35.7%) compared to non-responders (6.7%). Increased methylation in the *MECP2* promoter, alongside reduced expression following clozapine treatment in responders, was also reported (Kikuchi et al., 2021). Even within a monozygotic twin pair, where genetic background is controlled, the findings showed substantial clozapine-related methylation changes, underscoring the challenge of isolating TRS-specific epigenetic changes. Together, these observations also highlight the need for study designs with pre-treatment baselines to clarify whether such methylation differences represent underlying TRS mechanisms or are primarily treatment-induced.

## 4 Discussion

This scoping review summarized the current literature on the genetic architecture underlying TRS, examining how TRS has been identified and defined across studies and to integrate findings across multiple genomic domains. The included articles spanned GWAS, candidate genes, PRS, rare variants and CNVs, gene expression, and epigenetic research. Together, these findings suggest that TRS has a measurable, but complex and heterogeneous, genetic component and that key knowledge gaps must be addressed to advance mechanistic understanding and potential clinical translation.

### 4.1 Definition of TRS

Establishing the genetic architecture of any complex trait requires having a clear and consistent definition. The Treatment Response and Resistance in Psychosis working group developed consensus guidelines outlining minimum and optimal criteria for defining TRS across several clinical areas including current symptoms, adequacy of treatment, symptom domains, and time course (Howes et al., 2017). In practice, however, applying even the minimum consensus criteria in large-scale genetic studies may be time- and resource-intensive. This was reflected in the scoping review, where the definition and identification of TRS ranged from detailed clinical criteria to proxy measures such as clozapine use or polypharmacy. Nevertheless, across studies, most definitions of TRS converged on three criteria–failure to respond to antipsychotic trials (though the adequacy of such treatment was not always evaluated), persistence or worsening of symptoms over time, and the use of clozapine or polypharmacy. These shared criterions provide a starting point for harmonizing TRS definitions in future genetic research.

### 4.2 Definition of TRS and the potential for misclassification

An important limitation is the potential for treatment resistance misclassification due to substantial heterogeneity in how TRS was defined across studies. Clozapine use should be interpreted as an imperfect proxy for TRS, as its initiation is influenced by geographic and temporal variation in clinical practice; differences in access, monitoring requirements, local guidelines, and prescribing preferences may affect who receives clozapine and when, potentially introducing misclassification of the treatment-resistant phenotype. Using clozapine as a proxy for TRS may also exclude individuals with delayed access or barriers to clozapine treatment (leading to false negatives), while symptom persistence despite antipsychotic treatment could reflect partial response rather than true resistance (leading to false positives). This differential misclassification has important implications for power, effect size estimation, and the interpretation of null findings.

### 4.3 Selection of comparator groups

Selecting the appropriate comparison groups is equally important for interpreting genetic and biological findings. Comparing TRS to treatment-responsive schizophrenia can help identify mechanisms specifically related to non-response and treatment efficacy. Conversely, studies using only healthy controls contribute to our understanding of schizophrenia genetics more broadly, but not treatment resistance biology. Across studies, nearly 70% included individuals without TRS (non-TRS or treatment-responsive cases) as the comparator group.

Evidence from other psychiatric disorders support the distinction between genetic liability for illness risk and treatment response, with only partial overlap in conditions such as major depressive disorder (Fabbri et al., 2021). Future research should be deliberate and transparent in the selection of comparison groups and, where possible, include both treatment-responsive schizophrenia and healthy controls to clarify which differences are specific to treatment resistance.

### 4.4 Insights

Evidence from this scoping review indicates that the genetic architecture of TRS is only beginning to be delineated. More than 50% of the included studies focused on common genetic variants, yet findings explained a relatively small proportion of genetic liability for TRS. Most GWAS to date have concentrated on schizophrenia in general, rather than specific subsets, such as TRS. Consequently, TRS-specific PRS are still in early phases of development and existing work largely extrapolates from schizophrenia case-control PRS, which offers a rough approximation of treatment resistance risk. TRS and schizophrenia share a high genetic correlation as many of the genes and variants associated with TRS largely overlap with those for schizophrenia (Trubetskoy et al., 2022). In addition, clozapine-related outcomes, such as drug response or adverse effects, likely represent a secondary rather than direct genetic correlate of TRS. Resultant effects, such as neutropenia, are better understood as moderators of clozapine tolerability and treatment continuation. Given that clozapine is central to TRS management, genetic liability to neutropenia may still be highly clinically relevant but should be interpreted regarding safe clozapine use, rather than mechanisms of treatment resistance.

The included studies also pointed to a role for rare, high-impact genetic variation, but this area remains nascent. Rare variant analyses in TRS reported finding significantly greater burden of damaging variants in genes intolerant to loss-of-function or deleterious missense mutations, suggesting that the genome of severe and persistent refractory schizophrenia may carry highly penetrant variants, yet replication studies are needed. Most existing rare variant data come from exome-focused designs since systematic whole-genome sequencing efforts that integrate rare intronic and regulatory variants are largely absent. This represents a clear space for future research.

Transcriptomic and epigenomic data added further, yet still fragmented, insight into the genetic architecture of TRS. Differential gene expression profiles in peripheral and brain-derived tissues have been reported in TRS when compared with healthy controls, but specificity relative to treatment-responsive schizophrenia remains unclear. Similarly, TRS-associated differentially methylated loci often overlapped with methylation changes observed following clozapine initiation, raising the question of whether these epigenetic patterns index treatment resistance itself, cumulative drug effects, or a combination of both, highlighting the complexity of interpreting epigenetic signatures in this context.

Genetic pleiotropy with TRS comorbidities is an emerging area of investigation. Although schizophrenia exhibits genetic overlap with other conditions, current research assessing non-schizophrenia PRS in relation to TRS outcomes is sparse and largely non-significant. For instance, although polygenic liability for smoking initiation has been linked to schizophrenia risk, evidence for a robust or specific association with TRS remains limited (Peterson et al., 2021).

At the extreme end of the spectrum, ultra-TRS, where individuals fail to respond to clozapine and multiple augmentation strategies, has been understudied. Genetic, transcriptomic, and epigenomic data in this subgroup remains limited. This may reflect the rarity of ultra-TRS and the challenges associated with studying these individuals. Consequently, it remains unclear whether ultra-TRS reflects a quantitative extreme of the same liability spectrum or a qualitatively distinct subgroup with unique biological mechanisms. Addressing this question will require dedicated, well-powered studies of ultra-TRS, ideally incorporating longitudinal designs and multi-omic profiling.

A further limitation of the existing literature is the generally small sample sizes, with many studies including fewer than 200 participants. Null findings in underpowered studies may reflect false negatives rather than a true absence of association, while positive findings from small studies, particularly those lacking replication, should be interpreted cautiously due to an increased risk of false positives. Publication bias also cannot be excluded, as small candidate gene studies reporting positive findings may be more likely to be published than those reporting null results.

### 4.5 Ancestry and sex considerations

Most included articles were conducted in populations of predominantly European ancestry, with few modestly sized cohorts representing African and Asian populations. This likely reflects both the lower prevalence of TRS overall, which complicates recruitment of adequately powered multi-ancestry cohorts, and the methodological demands of TRS studies, including detailed longitudinal phenotyping, that concentrate research in high-income settings with established infrastructure. This imbalance has important analytical implications. Differences in population structure require careful control for population stratification within and across studies, typically through ancestry-informed design and adjustment to reduce the risk of confounding. Additionally, ancestry-specific differences in allele frequencies may affect the power to detect associations, particularly for low-frequency variants, and differences in linkage disequilibrium patterns can influence both locus discovery and the transferability of findings, including fine-mapping and polygenic prediction, across populations. Together, these limitations reduce the generalizability of current TRS genetic findings and hinder identification of ancestry-specific risk or protective factors. Broadening ancestral diversity in TRS research will be essential for developing more inclusive etiological models and for supporting equitable implementation of precision medicine.

Sex-specific characteristics in TRS are also underexplored in genetic research approaches. A small number of candidate gene studies reported sex-stratified associations, suggesting that genetic contributions to TRS may differ between males and females. Considering there are established sex-differences in prevalence, clinical presentation, and symptom severity within schizophrenia (Ferrer-Quintero et al., 2021; Li et al., 2016), integrating sex-stratified analyses in future TRS genetics studies may help clarify distinct clinical presentations of treatment-resistant phenotypes and differentiate TRS from treatment-responsive schizophrenia.

### 4.6 Limitations

This scoping review has limitations. First, article inclusion was restricted to English-language, which may have led to the omission of relevant studies published in other languages. Second, although the literature search was limited to PubMed, which includes both MEDLINE-indexed and non-MEDLINE biomedical literature, this may have reduced coverage of studies indexed exclusively in other databases. Third, substantial heterogeneity in TRS definitions, study designs, and outcome measures limited the extent to which findings could be quantitatively synthesized, though this variability reflects real-world clinical practice and provides insight into how TRS is operationalized across research. Lastly, as a scoping review, formal assessment of the methodological quality or risk of bias of individual studies was not conducted, and small, underpowered studies with positive findings may be overrepresented in the published literature.

### 4.7 Future directions

The available evidence indicates that TRS has a genetic architecture that both overlaps with and differs from that of schizophrenia risk. Rare variants and CNVs may be particularly relevant for a subset of individuals with severe or early-onset TRS, whereas common variant burden (as captured by schizophrenia PRS) appears to index genetic liability and illness severity rather than TRS-specific risk.

Despite increased attention in research, important questions remain. It is not yet clear whether TRS is genetically distinct from schizophrenia more broadly, or to what extent genetic markers reflect treatment resistance itself versus the effects of long-term antipsychotic exposure, illness severity and chronicity, or environmental stressors (e.g., trauma, substance use). Longitudinal evidence of gene expression and epigenetic variation in relation to treatment outcomes and symptom trajectories are also poorly understood.

Future research should prioritize large, ancestrally diverse TRS cohorts with standardized definitions aligned as closely as possible with consensus criteria (Howes et al., 2017). This could involve adopting stage-based or tiered TRS phenotypes (e.g., failure of two adequate antipsychotic trials, clozapine eligibility, clozapine non-response), clearly distinguishing treatment resistance from intolerance or non-adherence and applying *post hoc* phenotype mapping to harmonize heterogeneous cohorts for genetic analyses. Explicitly incorporating longitudinal designs with pre-treatment baselines will be essential for separating TRS trait markers from state effects and from molecular signatures driven by treatment, especially clozapine. Multi-omics integration approaches such as Mendelian randomisation, colocalisation analyses, transcriptome-wide association studies (TWAS), and gene-by-environment interaction models will be critical for distinguishing causal mechanisms from correlated signals. Whole-genome sequencing may further improve detection of rare variants associated with treatment resistance, potentially revealing novel biological pathways and therapeutic targets (Singh et al., 2024). Incorporating sex-stratified analyses and systematically capturing environmental exposures will also help clarify how genetic risk interacts with clinical and contextual factors to shape treatment outcomes for individuals with TRS.

### 4.8 Conclusion

This scoping review provides, to our knowledge, the first integrated overview of the genetics architecture of TRS across common variants, rare variants and CNVs, gene expression, and epigenetic studies. The findings highlight substantial complexity and heterogeneity in both TRS definitions and genetic results, with no single genetic profile emerging as definitive for TRS. Instead, TRS appears to arise from multiple, partly overlapping biological mechanisms, some shared with schizophrenia more broadly and others potentially specific to treatment resistance or clozapine response.

Future research incorporating trait-based phenotyping, longitudinal follow-up, and multi-omic approaches in diverse populations will be essential for refining the genetic architecture of TRS. A deeper understanding of TRS biology remains needed to meaningfully improve early identification of individuals at risk, optimize treatment strategies, and advance precision psychiatry for individuals living with schizophrenia.

## References

[B1] Alacam H. Akgun S. Akca H. Ozturk O. Kabukcu B. B. Herken H. (2016). miR-181b-5p, miR-195-5p and miR-301a-3p are related with treatment resistance in schizophrenia. Psychiatry Res. 245, 200–206. 10.1016/j.psychres.2016.08.037 27552670

[B2] Arranz M. J. Munro J. Owen M. J. Spurlock G. Sham P. C. Zhao J. (1998). Evidence for association between polymorphisms in the promoter and coding regions of the 5-HT2A receptor gene and response to clozapine. Mol. Psychiatry 3 (1), 61–66. 10.1038/sj.mp.4000348 9491814

[B3] Aytac H. M. Yazar M. S. Erol A. Pehlivan S. (2021). Investigation of inflammation related gene polymorphism of the mannose-binding lectin 2 in schizophrenia and bipolar disorder. Neurosciences 26 (4), 346–356. 10.17712/nsj.2021.4.20200050 34663707 PMC9037773

[B4] Aytac H. M. Ozdilli K. Tuncel F. C. Pehlivan M. Pehlivan S. (2022). Tumor necrosis factor-alpha (TNF-α) -238 G/A polymorphism is associated with the treatment resistance and attempted suicide in schizophrenia. Immunol. Invest 51 (2), 368–380. 10.1080/08820139.2020.1832115 33092426

[B5] Badrlou E. Ghafouri-Fard S. Omrani M. D. Neishabouri S. M. Arsang-Jang S. Taheri M. (2021). Expression of BDNF-associated lncRNAs in treatment-resistant schizophrenia patients. J. Mol. Neurosci. MN 71 (11), 2249–2259. 10.1007/s12031-020-01772-9 33403596

[B6] Bani-Fatemi A. Tasmim S. Graff A. Gerretsen P. Dada O. O. Kennedy J. L. (2019). The effect of ethnicity and immigration on treatment resistance in schizophrenia. Compr. Psychiatry 89, 28–32. 10.1016/j.comppsych.2018.12.003 30579127

[B7] Bilic P. Jukic V. Vilibic M. Savic A. Bozina N. (2014). Treatment-resistant schizophrenia and DAT and SERT polymorphisms. Gene 543 (1), 125–132. 10.1016/j.gene.2014.03.050 24680725

[B8] Bosia M. Lorenzi C. Pirovano A. Guglielmino C. Cocchi F. Spangaro M. (2015). COMT Val158Met and 5-HT1A-R -1019 C/G polymorphisms: effects on the negative symptom response to clozapine. Pharmacogenomics 16 (1), 35–44. 10.2217/pgs.14.150 25560469

[B9] Bozina N. Kuzman M. R. Medved V. Jovanovic N. Sertic J. Hotujac L. (2008). Associations between MDR1 gene polymorphisms and schizophrenia and therapeutic response to olanzapine in female schizophrenic patients. J. Psychiatr. Res. 42 (2), 89–97. 10.1016/j.jpsychires.2006.10.002 17113599

[B10] Casale A. D. Simmaco M. Modesti M. N. Zocchi C. Arena J. F. Bilotta I. (2023). DRD2, DRD3, and HTR2A single-nucleotide polymorphisms involvement in high treatment resistance to atypical antipsychotic drugs. Biomedicines 11 (7), 1–13. 10.3390/biomedicines11072088 PMC10377184 37509727

[B11] Cheng B. Cheng S. Li C. Wei W. Liu L. Meng P. (2023). Treatment resistance in schizophrenia is associated with attention deficit/hyperactivity disorder and gut microbiota: a genetic correlation and mendelian randomization study. Neuropsychobiology 82 (1), 24–32. 10.1159/000528316 36623478

[B12] Conley R. R. Kelly D. L. (2001). Management of treatment resistance in schizophrenia. Biol. Psychiatry 50 (11), 898–911. 10.1016/S0006-3223(01)01271-9 11743944

[B13] De Luca V. Chaudhary Z. Al-Chalabi N. Qian J. Borlido C. Gerretsen P. (2023). Genome-wide methylation analysis of treatment resistant schizophrenia. J. Neural Transm. 130 (2), 165–169. 10.1007/s00702-022-02585-3 36648581

[B14] Del Casale A. Gentile G. Lardani S. Modesti M. N. Arena J. F. Zocchi C. (2026). Investigating DRD2 and HTR2A polymorphisms in treatment-resistant schizophrenia: a comparative analysis with other treatment-resistant mental disorders and the healthy state. Eur. Arch. Psychiatry Clin. Neurosci. 276 (3), 1221–1231. 10.1007/s00406-025-01970-9 39934320 PMC13002770

[B15] Demirbugen O. M. Ozdemir F. Tok K. C. Dural E. Kir Y. Ulusoy M. (2023). The potential role of POR*28 and CYP1A2*F genetic variations and lifestyle factors on clozapine and N-DesmethylClozapine plasma levels in schizophrenia patients. Expert Opin. Drug Metab. Toxicol. 19 (5), 319–327. 10.1080/17425255.2023.2221849 37269349

[B16] Eap C. B. Bender S. Jaquenoud S. E. Cucchia G. Jonzier-Perey M. Baumann P. (2004). Nonresponse to clozapine and ultrarapid CYP1A2 activity: clinical data and analysis of CYP1A2 gene. J. Clin. Psychopharmacol. 24 (2), 214–219. 10.1097/01.jcp.0000116646.91923.2f 15206669

[B17] Escamilla R. Camarena B. Saracco-Alvarez R. Fresán A. Hernández S. Aguilar-García A. (2018). Association study between COMT, DRD2, and DRD3 gene variants and antipsychotic treatment response in Mexican patients with schizophrenia. Neuropsychiatr. Dis. Treat. 14, 2981–2987. 10.2147/NDT.S176455 30464483 PMC6223330

[B18] Fabbri C. Hagenaars S. P. John C. Williams A. T. Shrine N. Moles L. (2021). Genetic and clinical characteristics of treatment-resistant depression using primary care records in two UK cohorts. Mol. Psychiatry 26 (7), 3363–3373. 10.1038/s41380-021-01062-9 33753889 PMC8505242

[B19] Facal F. Costas J. (2025). Shared polygenic susceptibility to treatment response in severe affective and psychotic disorders: evidence from GWAS data sets. Prog. Neuropsychopharmacol. Biol. Psychiatry 136, 111183. 10.1016/j.pnpbp.2024.111183 39490915

[B20] Farrell M. Dietterich T. E. Harner M. K. Bruno L. M. Filmyer D. M. Shaughnessy R. A. (2023). Increased prevalence of rare copy number variants in treatment-resistant psychosis. Schizophr. Bull. 49 (4), 881–892. 10.1093/schbul/sbac175 36454006 PMC10318882

[B21] Ferrer-Quintero M. Green M. F. Horan W. P. Penn D. L. Kern R. S. Lee J. (2021). The effect of sex on social cognition and functioning in schizophrenia. Npj Schizophr. 7 (1), 57. 10.1038/s41537-021-00188-7 34853324 PMC8636592

[B22] Frank J. Lang M. Witt S. H. Strohmaier J. Rujescu D. Cichon S. (2015). Identification of increased genetic risk scores for schizophrenia in treatment-resistant patients. Mol. Psychiatry 20 (2), 150–151. 10.1038/mp.2014.56 24888364 PMC4356742

[B23] Funahashi Y. Yoshino Y. Iga J. I. Ueno S. I. (2023). Impact of clozapine on the expression of miR-675-3p in plasma exosomes derived from patients with schizophrenia. World J. Biol. Psychiatry Off. J. World Fed. Soc. Biol. Psychiatry 24 (4), 303–313. 10.1080/15622975.2022.2104924 35904423

[B24] Gasse C. Wimberley T. Wang Y. Mors O. Børglum A. Als T. D. (2019). Schizophrenia polygenic risk scores, urbanicity and treatment-resistant schizophrenia. Schizophr. Res. 212, 79–85. 10.1016/j.schres.2019.08.008 31447354

[B25] Griffiths K. Smart S. E. Barker G. J. Deakin B. Lawrie S. M. Lewis S. (2023). Treatment resistance NMDA receptor pathway polygenic score is associated with brain glutamate in schizophrenia. Schizophr. Res. 260, 152–159. 10.1016/j.schres.2023.08.020 37657282 PMC10873209

[B26] Hajj A. Obeid S. Sahyoun S. Haddad C. Azar J. Rabbaa Khabbaz L. (2019). Clinical and genetic factors associated with resistance to treatment in patients with schizophrenia: a case-control study. Int. J. Mol. Sci. 20 (19), 4753. 10.3390/ijms20194753 31557839 PMC6801865

[B27] Hannon E. Dempster E. L. Mansell G. Burrage J. Bass N. Bohlken M. M. (2021). DNA methylation meta-analysis reveals cellular alterations in psychosis and markers of treatment-resistant schizophrenia. Elife 10, e58430. 10.7554/eLife.58430 33646943 PMC8009672

[B28] Hong C. J. Yu Y. W. Lin C. H. Song H. L. Lai H. C. Yang K. H. (2000). Association study of apolipoprotein E epsilon4 with clinical phenotype and clozapine response in schizophrenia. Neuropsychobiology 42 (4), 172–174. 10.1159/000026689 11096331

[B29] Hong C. J. Yu Y. W. Y. Lin C. H. Tsai S. J. (2003). An association study of a brain-derived neurotrophic factor Val66Met polymorphism and clozapine response of schizophrenic patients. Neurosci. Lett. 349 (3), 206–208. 10.1016/s0304-3940(03)00828-0 12951204

[B30] Hotta Y. Ohnuma T. Hanzawa R. Shibata N. Maeshima H. Baba H. (2011). Association study between Disrupted-in-Schizophrenia-1 (DISC1) and Japanese patients with treatment-resistant schizophrenia (TRS). Prog. Neuropsychopharmacol. Biol. Psychiatry 35 (2), 636–639. 10.1016/j.pnpbp.2011.01.011 21256178

[B31] Howes O. D. McCutcheon R. Agid O. de Bartolomeis A. van Beveren N. J. M. Birnbaum M. L. (2017). Treatment-resistant schizophrenia: treatment response and resistance in psychosis (TRRIP) working group consensus guidelines on diagnosis and terminology. Am. J. Psychiatry 174 (3), 216–229. 10.1176/appi.ajp.2016.16050503 27919182 PMC6231547

[B32] Hwang R. Shinkai T. De Luca V. Müller D. J. Ni X. Macciardi F. (2005). Association study of 12 polymorphisms spanning the dopamine D(2) receptor gene and clozapine treatment response in two treatment refractory/intolerant populations. Psychopharmacol. Berl. 181 (1), 179–187. 10.1007/s00213-005-2223-5 15830237

[B33] Inada T. Nakamura A. Iijima Y. (2003). Relationship between catechol-O-methyltransferase polymorphism and treatment-resistant schizophrenia. Am. J. Med. Genet. Part B Neuropsychiatr. Genet. Off. Publ. Int. Soc. Psychiatr. Genet. 120B (1), 35–39. 10.1002/ajmg.b.20023 12815736

[B34] Jia P. Jayathilake K. Zhao Z. Meltzer H. Y. (2011). Association of FAS, a TNF-α receptor gene, with treatment resistant schizophrenia. Schizophr. Res. 129 (2-3), 211–212. 10.1016/j.schres.2011.04.013 21549565

[B35] Ji X. Takahashi N. Branko A. Ishihara R. Nagai T. Mouri A. (2008a). An association between serotonin receptor 3B gene (HTR3B) and treatment-resistant schizophrenia (TRS) in a Japanese population. Nagoya J. Med. Sci. 70 (1-2), 11–17. 18807291

[B36] Ji X. Takahashi N. Saito S. Ishihara R. Maeno N. Inada T. (2008b). Relationship between three serotonin receptor subtypes (HTR3A, HTR2A and HTR4) and treatment-resistant schizophrenia in the Japanese population. Neurosci. Lett. 435 (2), 95–98. 10.1016/j.neulet.2008.01.083 18359159

[B37] Kane J. Honigfeld G. Singer J. Meltzer H. (1988). Clozapine for the treatment-resistant schizophrenic: a double-blind comparison with chlorpromazine. Arch. Gen. Psychiatry 45 (9), 789–796. 10.1001/archpsyc.1988.01800330013001 3046553

[B38] Kappel D. B. Legge S. E. Hubbard L. Willcocks I. R. O'Connell K. S. Smith R. L. (2023). Genomic stratification of clozapine prescription patterns using schizophrenia polygenic scores. Biol. Psychiatry 93 (2), 149–156. 10.1016/j.biopsych.2022.07.014 36244804 PMC10804961

[B39] Kappel D. B. Rees E. Fenner E. King A. Jansen J. Helthuis M. (2024). Rare variants in pharmacogenes influence clozapine metabolism in individuals with schizophrenia. Eur. Neuropsychopharmacol. J. Eur. Coll. Neuropsychopharmacol. 80, 47–54. 10.1016/j.euroneuro.2023.12.007 PMC7619122 38310750

[B40] Kennedy J. L. Altar C. A. Taylor D. L. Degtiar I. Hornberger J. C. (2014). The social and economic burden of treatment-resistant schizophrenia: a systematic literature review. Int. Clin. Psychopharmacol. 29 (2), 63–76. 10.1097/YIC.0b013e32836508e6 23995856

[B41] Kiltschewskij D. J. Reay W. R. Geaghan M. P. Atkins J. R. Xavier A. Zhang X. (2024). Alteration of DNA methylation and epigenetic scores associated with features of schizophrenia and common variant genetic risk. Biol. Psychiatry 95 (7), 647–661. 10.1016/j.biopsych.2023.07.010 37480976

[B42] Kikuchi M. Nakazawa T. Kinoshita M. Yamamori H. Yasuda Y. Fujimoto M. (2021). Methylation analysis in monozygotic twins with treatment-resistant schizophrenia and discordant responses to clozapine. Front. Psychiatry 12, 734606. 10.3389/fpsyt.2021.734606 34616320 PMC8488120

[B43] Kluge M. Schuld A. Schacht A. Himmerich H. Dalal M. A. Wehmeier P. M. (2009). Effects of clozapine and olanzapine on cytokine systems are closely linked to weight gain and drug-induced fever. Psychoneuroendocrinology 34 (1), 118–128. 10.1016/j.psyneuen.2008.08.016 18835660

[B44] Koga A. Bani-Fatemi A. Hettige N. Borlido C. Zai C. Strauss J. (2017). GWAS analysis of treatment resistant schizophrenia: interaction effect of childhood trauma. Pharmacogenomics 18 (7), 663–671. 10.2217/pgs-2016-0137 28453389

[B45] Kogure M. Kanahara N. Miyazawa A. Oishi K. Nakata Y. Oda Y. (2021). Interacting roles of COMT and GAD1 genes in patients with treatment-resistant schizophrenia: a genetic association study of schizophrenia patients and healthy controls. J. Mol. Neurosci. MN 71 (12), 2575–2582. 10.1007/s12031-021-01866-y 34125398

[B46] Kohlrausch F. B. Gama C. S. Lobato M. I. Belmonte-de-Abreu P. Callegari-Jacques S. M. Gesteira A. (2008). Naturalistic pharmacogenetic study of treatment resistance to typical neuroleptics in European-Brazilian schizophrenics. Pharmacogenet Genomics 18 (7), 599–609. 10.1097/FPC.0b013e328301a763 18551040

[B47] Kondo T. Mihara K. Suzuki A. Yasui-Furukori N. Kaneko S. (2003). Combination of dopamine D2 receptor gene polymorphisms as a possible predictor of treatment-resistance to dopamine antagonists in schizophrenic patients. Prog. Neuropsychopharmacol. Biol. Psychiatry 27 (6), 921–926. 10.1016/S0278-5846(03)00151-9 14499308

[B48] Konte B. Walters J. T. R. Rujescu D. Legge S. E. Pardiñas A. F. Cohen D. (2021). HLA-DQB1 6672G>C (rs113332494) is associated with clozapine-induced neutropenia and agranulocytosis in individuals of European ancestry. Transl. Psychiatry 11 (1), 214. 10.1038/s41398-021-01322-w 33846298 PMC8042025

[B49] Kowalec K. Lu Y. Sariaslan A. Song J. Ploner A. Dalman C. (2021). Increased schizophrenia family history burden and reduced premorbid IQ in treatment-resistant schizophrenia: a Swedish national register and genomic study. Mol. Psychiatry 26 (8), 4487–4495. 10.1038/s41380-019-0575-1 31712719 PMC9731609

[B50] Krzystanek M. Asman M. Witecka J. Pałasz A. Wiaderkiewicz R. (2021). Selected single-nucleotide variants in GRIN1, GRIN2A, and GRIN2B encoding subunits of the NMDA receptor are not biomarkers of schizophrenia resistant to clozapine: exploratory study. Pharmacol. Rep. P. R. 73 (1), 309–315. 10.1007/s43440-020-00165-4 33025395 PMC7862503

[B51] Kushima I. Aleksic B. Nakatochi M. Shimamura T. Shiino T. Yoshimi A. (2017). High-resolution copy number variation analysis of schizophrenia in Japan. Mol. Psychiatry 22 (3), 430–440. 10.1038/mp.2016.88 27240532

[B52] Land R. Siskind D. McArdle P. Kisely S. Winckel K. Hollingworth S. A. (2017). The impact of clozapine on hospital use: a systematic review and meta-analysis. Acta Psychiatr. Scand. 135 (4), 296–309. 10.1111/acps.12700 28155220

[B53] Legge S. E. Hamshere M. L. Ripke S. Pardinas A. F. Goldstein J. I. Rees E. (2017). Genome-wide common and rare variant analysis provides novel insights into clozapine-associated neutropenia. Mol. Psychiatry 22 (10), 1502–1508. 10.1038/mp.2016.97 27400856 PMC5065090

[B54] Legge S. E. Pardiñas A. F. Helthuis M. Jansen J. A. Jollie K. Knapper S. (2019). A genome-wide association study in individuals of African ancestry reveals the importance of the Duffy-null genotype in the assessment of clozapine-related neutropenia. Mol. Psychiatry 24 (3), 328–337. 10.1038/s41380-018-0335-7 30647433

[B55] Lenk H. Ç. Koch E. O’Connell K. S. Smith R. L. Akkouh I. A. Djurovic S. (2024). Genome-wide association analysis of treatment resistant schizophrenia for variant discovery and polygenic assessment. Hum. Genomics 18 (1), 108. 10.1186/s40246-024-00673-x 39334510 PMC11438281

[B56] Li J. Meltzer H. Y. (2014). A genetic locus in 7p12.2 associated with treatment resistant schizophrenia. Schizophr. Res. 159 (2-3), 333–339. 10.1016/j.schres.2014.08.018 25223841

[B57] Li R. Ma X. Wang G. Yang J. Wang C. (2016). Why sex differences in schizophrenia? J. Transl. Neurosci. 1 (1), 37–42. 29152382 PMC5688947

[B58] Lim K. Yee J. Y. See Y. M. Ng B. T. Zheng S. Tang C. (2023). Deconstructing the genetic architecture of treatment-resistant schizophrenia in East Asian ancestry. Asian J. Psychiatry 90, 103826. 10.1016/j.ajp.2023.103826 37944474

[B59] Liu C. Bousman C. A. Pantelis C. Skafidas E. Zhang D. Yue W. (2017). Pathway-wide association study identifies five shared pathways associated with schizophrenia in three ancestral distinct populations. Transl. Psychiatry 7 (2), e1037. 10.1038/tp.2017.8 28221366 PMC5438037

[B60] Lu A. K. M. Lin J. J. Tseng H. H. Wang X. Y. Jang F. L. Chen P. S. (2023). DNA methylation signature aberration as potential biomarkers in treatment-resistant schizophrenia: constructing a methylation risk score using a machine learning method. J. Psychiatr. Res. 157, 57–65. 10.1016/j.jpsychires.2022.11.008 36442407

[B62] Martin A. K. Mowry B. (2016). Increased rare duplication burden genomewide in patients with treatment-resistant schizophrenia. Psychol. Med. 46 (3), 469–476. 10.1017/S0033291715001701 26349998

[B63] Masuda T. Misawa F. Takase M. Kane J. M. Correll C. U. (2019). Association with hospitalization and all-cause discontinuation among patients with schizophrenia on clozapine vs other oral second-generation antipsychotics: a systematic review and meta-analysis of cohort studies. JAMA Psychiatry 76 (10), 1052–1062. 10.1001/jamapsychiatry.2019.1702 31365048 PMC6669790

[B64] Maury E. A. Sherman M. A. Genovese G. Gilgenast T. G. Kamath T. Burris S. J. (2023). Schizophrenia-associated somatic copy-number variants from 12,834 cases reveal recurrent *NRXN1* and *ABCB11* disruptions. Cell Genomics 3 (8), 100356. 10.1016/j.xgen.2023.100356 37601975 PMC10435376

[B65] Menus Á. Kiss Á. Tóth K. Sirok D. Déri M. Fekete F. (2020). Association of clozapine-related metabolic disturbances with CYP3A4 expression in patients with schizophrenia. Sci. Rep. 10 (1), 21283. 10.1038/s41598-020-78474-0 33277605 PMC7718230

[B66] Millgate E. Hide O. Lawrie S. M. Murray R. M. MacCabe J. H. Kravariti E. (2022). Neuropsychological differences between treatment-resistant and treatment-responsive schizophrenia: a meta-analysis. Psychol. Med. 52 (1), 1–13. 10.1017/S0033291721004128 36415088 PMC8711103

[B67] Miyazawa A. Kanahara N. Kogure M. Otsuka I. Okazaki S. Watanabe Y. (2022). A preliminary genetic association study of GAD1 and GABAB receptor genes in patients with treatment-resistant schizophrenia. Mol. Biol. Rep. 49 (3), 2015–2024. 10.1007/s11033-021-07019-z 34845648

[B68] Mohamed Saini S. Bousman C. A. Mancuso S. G. Cropley V. Van Rheenen T. E. Lenroot R. K. (2023). Genetic variation in glutamatergic genes moderates the effects of childhood adversity on brain volume and IQ in treatment-resistant schizophrenia. Schizophrenia 9 (1), 59. 10.1038/s41537-023-00381-w 37709784 PMC10502098

[B69] Moretti P. N. Ota V. K. Gouvea E. S. Pedrini M. Santoro M. L. Talarico F. (2018). Accessing gene expression in treatment-resistant schizophrenia. Mol. Neurobiol. 55 (8), 7000–7008. 10.1007/s12035-018-0876-4 29374346

[B70] Morimoto Y. Ono S. Yoshida S. Mishima H. Kinoshita A. Tanaka T. (2021). A unique missense variant in the E1A-binding protein P400 gene is implicated in schizophrenia by whole-exome sequencing and mutant mouse models. Transl. Psychiatry 11 (1), 132. 10.1038/s41398-021-01258-1 33602898 PMC7892873

[B71] Mostaid M. S. Lee T. T. Chana G. Sundram S. Shannon Weickert C. Pantelis C. (2017). Peripheral transcription of NRG-ErbB pathway genes are upregulated in treatment-resistant schizophrenia. Front. Psychiatry 8, 225. 10.3389/fpsyt.2017.00225 29163244 PMC5681734

[B72] Mostaid M. S. Pantelis C. Everall I. P. Bousman C. A. (2018). Decreased peripheral TNF alpha (*TNF-α*) mRNA expression in patients with treatment-resistant schizophrenia. Schizophr. Res. 202, 387–388. 10.1016/j.schres.2018.04.032 29706448

[B73] Mouaffak F. Kebir O. Chayet M. Tordjman S. Vacheron M. N. Millet B. (2011a). Association of disrupted in schizophrenia 1 (DISC1) missense variants with ultra-resistant schizophrenia. Pharmacogenomics J. 11 (4), 267–273. 10.1038/tpj.2010.40 20531374

[B74] Mouaffak F. Kebir O. Bellon A. Gourevitch R. Tordjman S. Viala A. (2011b). Association of an UCP4 (SLC25A27) haplotype with ultra-resistant schizophrenia. Pharmacogenomics 12 (2), 185–193. 10.2217/pgs.10.179 21332312

[B61] Naveen M. Patil A. N. Pattanaik S. Kaur A. Banerjee D. Grover S. (2020). ABCB1 and DRD3 polymorphism as a response predicting biomarker and tool for pharmacogenetically guided clozapine dosing in Asian Indian treatment resistant schizophrenia patients. Asian J. Psychiatry 48, 101918. 10.1016/j.ajp.2019.101918 31896438

[B75] Nakata Y. Kanahara N. Kimura A. Niitsu T. Komatsu H. Oda Y. (2021). Oxytocin system dysfunction in patients with treatment-resistant schizophrenia: alterations of blood oxytocin levels and effect of a genetic variant of OXTR. J. Psychiatr. Res. 138, 219–227. 10.1016/j.jpsychires.2021.03.053 33866050

[B76] Nakazawa T. Kikuchi M. Ishikawa M. Yamamori H. Nagayasu K. Matsumoto T. (2017). Differential gene expression profiles in neurons generated from lymphoblastoid B-cell line-derived iPS cells from monozygotic twin cases with treatment-resistant schizophrenia and discordant responses to clozapine. Schizophr. Res. 181, 75–82. 10.1016/j.schres.2016.10.012 28277309

[B77] NCBI (2026a). Optimization of clozapine treatment: study of variables affecting response in Uruguayan patients with schizophrenia - PubMed. Available online at: https://pubmed-ncbi-nlm-nih-gov.uml.idm.oclc.org/39714785/ (Accessed April 10, 2026). 10.1097/JCP.0000000000001933 39714785

[B78] NCBI (2026b). Potential role of patients’ CYP3A-Status in clozapine pharmacokinetics - PubMed. Available online at: https://pubmed-ncbi-nlm-nih-gov.uml.idm.oclc.org/28340122/ (Accessed April 10, 2026). 10.1093/ijnp/pyx019 PMC5492788 28340122

[B79] Oishi K. Kanahara N. Takase M. Oda Y. Nakata Y. Niitsu T. (2018). Vulnerable combinations of functional dopaminergic polymorphisms to late-onset treatment resistant schizophrenia. PloS One 13 (11), e0207133. 10.1371/journal.pone.0207133 30408108 PMC6224074

[B80] Okhuijsen-Pfeifer C. van der Horst M. Z. Bousman C. A. Lin B. van Eijk K. R. Ripke S. (2022). Genome-wide association analyses of symptom severity among clozapine-treated patients with schizophrenia spectrum disorders. Transl. Psychiatry 12 (1), 145. 10.1038/s41398-022-01884-3 35393395 PMC8989876

[B81] Ota V. K. Spíndola L. N. Gadelha A. dos Santos Filho A. F. Santoro M. L. Christofolini D. M. (2012). DRD1 rs4532 polymorphism: a potential pharmacogenomic marker for treatment response to antipsychotic drugs. Schizophr. Res. 142 (1-3), 206–208. 10.1016/j.schres.2012.08.003 23036699

[B82] Ozdemir F. Oz M. D. Tok K. C. Dural E. Kır Y. Gumustas M. (2025). The effects of UGT1A4 and ABCB1 polymorphisms on clozapine and N- desmethyl clozapine plasma levels in Turkish schizophrenia patients. Toxicol. Appl. Pharmacol. 495, 117219. 10.1016/j.taap.2024.117219 39761923

[B83] O’Connell K. S. Koch E. Lenk H. Ç. Akkouh I. A. Hindley G. Jaholkowski P. (2023). Polygenic overlap with body-mass index improves prediction of treatment-resistant schizophrenia. Psychiatry Res. 325, 115217. 10.1016/j.psychres.2023.115217 37146461 PMC10788293

[B84] Pae C. U. Serretti A. Artioli P. Kim T. S. Kim J. J. Lee C. U. (2006). Interaction analysis between 5-HTTLPR and TNFA -238/-308 polymorphisms in schizophrenia. J. Neural Transm. 113 (7), 887–897. 10.1007/s00702-005-0358-5 16252073

[B85] Pardiñas A. F. Nalmpanti M. Pocklington A. J. Legge S. E. Medway C. King A. (2019). Pharmacogenomic variants and drug interactions identified through the genetic analysis of clozapine metabolism. Am. J. Psychiatry 176 (6), 477–486. 10.1176/appi.ajp.2019.18050589 30922102

[B86] Pardiñas A. F. Smart S. E. Willcocks I. R. Holmans P. A. Dennison C. A. Lynham A. J. (2022). Interaction testing and polygenic risk scoring to estimate the association of common genetic variants with treatment resistance in schizophrenia. JAMA Psychiatry 79 (3), 260–269. 10.1001/jamapsychiatry.2021.3799 35019943 PMC8756361

[B87] Pardiñas A. F. Kappel D. B. Roberts M. Tipple F. Shitomi-Jones L. M. King A. (2023). Pharmacokinetics and pharmacogenomics of clozapine in an ancestrally diverse sample: a longitudinal analysis and genome-wide association study using UK clinical monitoring data. Lancet Psychiatry 10 (3), 209–219. 10.1016/S2215-0366(23)00002-0 36804072 PMC10824469

[B88] Patil A. N. Kasudhan K. S. Naveen M. Batra G. K. Chakrabarti S. Avasthi A. (2021). Precise pharmacogenetic pharmacometabolomic (PPP) guided clozapine therapy in treatment resistant schizophrenia: insights from one ethnicity experiment. Schizophr. Res. 237, 26–28. 10.1016/j.schres.2021.08.016 34481201

[B89] Pérez-Rodríguez D. Penedo M. A. Rivera-Baltanás T. Peña-Centeno T. Burkhardt S. Fischer A. (2023). MiRNA differences related to treatment-resistant schizophrenia. Int. J. Mol. Sci. 24 (3), 1891. 10.3390/ijms24031891 36768211 PMC9916039

[B90] Peterson R. E. Bigdeli T. B. Ripke S. Bacanu S. A. Gejman P. V. Levinson D. F. (2021). Genome-wide analyses of smoking behaviors in schizophrenia: findings from the psychiatric genomics consortium. J. Psychiatr. Res. 137, 215–224. 10.1016/j.jpsychires.2021.02.027 33691233 PMC8096167

[B91] Piatkov I. Caetano D. Assur Y. Lau S. L. Coelho M. Jones T. (2017). CYP2C19*17 protects against metabolic complications of clozapine treatment. World J. Biol. Psychiatry Off. J. World Fed. Soc. Biol. Psychiatry 18 (7), 521–527. 10.1080/15622975.2017.1347712 28664816

[B92] Pinheiro D. S. Santos R. da S. de Brito R. B. Cruz A. H. da S. Ghedini P. C. Reis A. A. S. (2017). GSTM1/GSTT1 double-null genotype increases risk of treatment-resistant schizophrenia: a genetic association study in Brazilian patients. PloS One 12 (8), e0183812. 10.1371/journal.pone.0183812 28837637 PMC5570380

[B93] Rajagopal V. M. Rajkumar A. P. Jacob K. S. Jacob M. (2018). Gene-gene interaction between DRD4 and COMT modulates clinical response to clozapine in treatment-resistant schizophrenia. Pharmacogenet Genomics. 28 (1), 31–35. 10.1097/FPC.0000000000000314 29087970

[B94] Rajkumar A. P. Poonkuzhali B. Kuruvilla A. Srivastava A. Jacob M. Jacob K. S. (2012). Outcome definitions and clinical predictors influence pharmacogenetic associations between HTR3A gene polymorphisms and response to clozapine in patients with schizophrenia. Psychopharmacol. Berl. 224 (3), 441–449. 10.1007/s00213-012-2773-2 22700043

[B95] Räsänen N. Tiihonen J. Koskuvi M. Trontti K. Cheng L. Hill A. F. (2025). miRNA profiling of hiPSC-derived neurons from monozygotic twins discordant for schizophrenia. Schizophrenia 11 (1), 21. 10.1038/s41537-025-00573-6 39966401 PMC11836399

[B96] Ruderfer D. M. Charney A. W. Readhead B. Kidd B. A. Kähler A. K. Kenny P. J. (2016). Polygenic overlap between schizophrenia risk and antipsychotic response: a genomic medicine approach. Lancet Psychiatry 3 (4), 350–357. 10.1016/S2215-0366(15)00553-2 26915512 PMC4982509

[B97] Sagud M. Tudor L. Uzun S. Perkovic M. N. Zivkovic M. Konjevod M. (2018). Haplotypic and genotypic association of Catechol-O-Methyltransferase rs4680 and rs4818 polymorphisms and treatment resistance in schizophrenia. Front. Pharmacol. 9, 705. 10.3389/fphar.2018.00705 30018555 PMC6037851

[B98] Saha S. Chant D. Welham J. McGrath J. (2005). A systematic review of the prevalence of schizophrenia. PLoS Med. 2 (5), e141. 10.1371/journal.pmed.0020141 15916472 PMC1140952

[B99] Singh G. Rajan-Babu I. S. Carrion P. Rowell W. Rogic S. Pour M. G. (2024). High yield of deep phenotyping and long read whole genome sequencing in treatment-resistant psychosis. Eur. Neuropsychopharmacol. 87, 193. 10.1016/j.euroneuro.2024.08.384

[B100] Solmi M. Seitidis G. Mavridis D. Correll C. U. Dragioti E. Guimond S. (2023). Incidence, prevalence, and global burden of schizophrenia - data, with critical appraisal, from the global burden of disease (GBD) 2019. Mol. Psychiatry 28 (12), 5319–5327. 10.1038/s41380-023-02138-4 37500825

[B101] Souza R. P. de Luca V. Meltzer H. Y. Lieberman J. A. Kennedy J. L. (2010). Influence of serotonin 3A and 3B receptor genes on clozapine treatment response in schizophrenia. Pharmacogenet Genomics 20 (4), 274–276. 10.1097/FPC.0b013e328337ce3e 20168265

[B102] Stroup T. S. Gerhard T. Crystal S. Huang C. Olfson M. (2016). Comparative effectiveness of clozapine and standard antipsychotic treatment in adults with schizophrenia. Am. J. Psychiatry 173 (2), 166–173. 10.1176/appi.ajp.2015.15030332 26541815

[B103] Sullivan P. F. Kendler K. S. Neale M. C. (2003). Schizophrenia as a complex trait: evidence from a meta-analysis of twin studies. Arch. Gen. Psychiatry 60 (12), 1187–1192. 10.1001/archpsyc.60.12.1187 14662550

[B104] Sun J. Zhang X. Cong Q. Chen D. Yi Z. Huang H. (2022). miR143-3p–Mediated NRG-1–Dependent mitochondrial dysfunction contributes to olanzapine resistance in refractory schizophrenia. Biol. Psychiatry 92 (5), 419–433. 10.1016/j.biopsych.2022.03.012 35662508

[B105] Taheri N. Pirboveiri R. Sayyah M. Bijanzadeh M. Ghandil P. (2023). Association of DRD2, DRD4 and COMT genes variants and their gene-gene interactions with antipsychotic treatment response in patients with schizophrenia. BMC Psychiatry 23 (1), 781. 10.1186/s12888-023-05292-9 37880658 PMC10599059

[B106] Takao T. Tachikawa H. Kawanishi Y. Katano T. Sen B. Homma M. (2006). Association of treatment-resistant schizophrenia with the G2677A/T and C3435T polymorphisms in the ATP-binding cassette subfamily B member 1 gene. Psychiatr. Genet. 16 (2), 47–48. 10.1097/01.ypg.0000194441.04684.db 16538177

[B107] Talarico F. Costa G. O. Ota V. K. Santoro M. L. Noto C. Gadelha A. (2022). Systems-level analysis of genetic variants reveals functional and spatiotemporal context in treatment-resistant schizophrenia. Mol. Neurobiol. 59 (5), 3170–3182. 10.1007/s12035-022-02794-7 35278208

[B108] Taylor D. L. Tiwari A. K. Lieberman J. A. Potkin S. G. Meltzer H. Y. Knight J. (2016). Genetic association analysis of N-methyl-D-aspartate receptor subunit gene GRIN2B and clinical response to clozapine. Hum. Psychopharmacol. 31 (2), 121–134. 10.1002/hup.2519 26876050

[B109] Taylor D. L. Tiwari A. K. Lieberman J. A. Potkin S. G. Meltzer H. Y. Knight J. (2017). Pharmacogenetic analysis of functional glutamate system gene variants and clinical response to clozapine. Mol. Neuropsychiatry 2 (4), 185–197. 10.1159/000449224 28277565 PMC5318922

[B110] Teo C. Zai C. Borlido C. Tomasetti C. Strauss J. Shinkai T. (2012). Analysis of treatment-resistant schizophrenia and 384 markers from candidate genes. Pharmacogenet Genomics 22 (11), 807–811. 10.1097/FPC.0b013e3283586c04 23047292

[B111] Terzić T. Kastelic M. Dolžan V. Plesničar B. K. (2015a). Genetic variability testing of neurodevelopmental genes in schizophrenic patients. J. Mol. Neurosci. MN 56 (1), 205–211. 10.1007/s12031-014-0482-5 25529856

[B112] Terzić T. Kastelic M. Dolžan V. Plesničar B. K. (2015b). Influence of 5-HT1A and 5-HTTLPR genetic variants on the schizophrenia symptoms and occurrence of treatment-resistant schizophrenia. Neuropsychiatr. Dis. Treat. 11, 453–459. 10.2147/NDT.S76494 25759587 PMC4345972

[B113] Terzić T. Kastelic M. Dolžan V. Plesničar B. K. (2016). Genetic polymorphisms in dopaminergic system and treatment-resistant schizophrenia. Psychiatr. Danub 28 (2), 127–131. 27287786

[B114] The Network and Pathway Analysis Subgroup of the Psychiatric Genomics Consortium (2015). Psychiatric genome-wide association study analyses implicate neuronal, immune and histone pathways. Nat. Neurosci. 18 (2), 199–209. 10.1038/nn.3922 25599223 PMC4378867

[B115] Tiihonen J. Mittendorfer-Rutz E. Majak M. Mehtälä J. Hoti F. Jedenius E. (2017). Real-world effectiveness of antipsychotic treatments in a nationwide cohort of 29 823 patients with schizophrenia. JAMA Psychiatry 74 (7), 686–693. 10.1001/jamapsychiatry.2017.1322 28593216 PMC5710250

[B116] Tricco A. C. Lillie E. Zarin W. O'Brien K. K. Colquhoun H. Levac D. (2018). PRISMA extension for scoping reviews (PRISMA-ScR): checklist and explanation. Ann. Intern Med. 169 (7), 467–473. 10.7326/M18-0850 30178033

[B117] Trubetskoy V. Pardiñas A. F. Qi T. Panagiotaropoulou G. Awasthi S. Bigdeli T. B. (2022). Mapping genomic loci implicates genes and synaptic biology in schizophrenia. Nature 604 (7906), 502–508. 10.1038/s41586-022-04434-5 35396580 PMC9392466

[B118] Tsai S. J. Wang Y. C. Yu Younger W. Y. Lin C. H. Yang K. H. Hong C. J. (2001). Association analysis of polymorphism in the promoter region of the alpha2a-adrenoceptor gene with schizophrenia and clozapine response. Schizophr. Res. 49 (1-2), 53–58. 10.1016/s0920-9964(00)00127-4 11343863

[B119] Werner M. C. F. Wirgenes K. V. Haram M. Bettella F. Lunding S. H. Rødevand L. (2020). Indicated association between polygenic risk score and treatment-resistance in a naturalistic sample of patients with schizophrenia spectrum disorders. Schizophr. Res. 218, 55–62. 10.1016/j.schres.2020.03.006 32171635

[B120] Willcocks I. R. Legge S. E. Nalmpanti M. Mazzeo L. King A. Jansen J. (2021). Clozapine metabolism is associated with absolute neutrophil count in individuals with treatment-resistant schizophrenia. Front. Pharmacol. 12, 658734. 10.3389/fphar.2021.658734 33959025 PMC8094024

[B121] Wimberley T. Støvring H. Sørensen H. J. Horsdal H. T. MacCabe J. H. Gasse C. (2016). Predictors of treatment resistance in patients with schizophrenia: a population-based cohort study. Lancet Psychiatry 3 (4), 358–366. 10.1016/S2215-0366(15)00575-1 26922475

[B122] Wimberley T. MacCabe J. H. Laursen T. M. Sørensen H. J. Astrup A. Horsdal H. T. (2017). Mortality and self-harm in association with clozapine in treatment-resistant schizophrenia. Am. J. Psychiatry 174 (10), 990–998. 10.1176/appi.ajp.2017.16091097 28750580

[B123] Xiong Y. Krebs K. Jermy B. Karlsson R. Pasman J. A. Nguyen T. D. (2025). Genome-wide association meta-analysis and rare copy number variant analysis of treatment-resistant depression. Mol. Psychiatry 30 (11), 5024–5033. 10.1038/s41380-025-03084-z 40571737 PMC12532596

[B124] Xu X. Xie S. Shi X. Lv J. Tang X. Wang X. (2015). Hexanucleotide repeat expansion in C9ORF72 is not detected in the treatment-resistant schizophrenia patients of Chinese Han. PloS One 10 (12), e0145347. 10.1371/journal.pone.0145347 26691640 PMC4687052

[B125] You X. Zhang Y. Long Q. Liu Z. Ma X. Lu Z. (2020). Investigating aberrantly expressed microRNAs in peripheral blood mononuclear cells from patients with treatment-resistant schizophrenia using miRNA sequencing and integrated bioinformatics. Mol. Med. Rep. 22 (5), 4340–4350. 10.3892/mmr.2020.11513 33000265 PMC7533444

[B126] Zazueta A. Castillo T. Cavieres Á. González R. Abarca M. Nieto R. R. (2022). Polymorphisms in schizophrenia-related genes are potential predictors of antipsychotic treatment resistance and refractoriness. Int. J. Neuropsychopharmacol. 25 (9), 701–708. 10.1093/ijnp/pyac025 35416253 PMC9515128

[B127] Zhang J. P. Lencz T. Geisler S. DeRosse P. Bromet E. J. Malhotra A. K. (2013). Genetic variation in BDNF is associated with antipsychotic treatment resistance in patients with schizophrenia. Schizophr. Res. 146 (1-3), 285–288. 10.1016/j.schres.2013.01.020 23433505 PMC3622803

[B128] Zoghbi A. W. Dhindsa R. S. Goldberg T. E. Mehralizade A. Motelow J. E. Wang X. (2021). High-impact rare genetic variants in severe schizophrenia. Proc. Natl. Acad. Sci. U. S. A. 118 (51), e2112560118. 10.1073/pnas.2112560118 34903660 PMC8713775

